# Telenovela: an innovative colorectal cancer screening health messaging tool

**DOI:** 10.3402/ijch.v72i0.21301

**Published:** 2013-08-05

**Authors:** Melany Cueva, Regina Kuhnley, Jozieta Slatton, Mark Dignan, Emily Underwood, Kate Landis

**Affiliations:** 1 Community Health Aide Program, Alaska Native Tribal Health Consortium, Anchorage, AK, USA; 2 Arctic Slope Native Association, Barrow, AK, USA; 3 Department of Internal Medicine, University of Kentucky, Lexington, KY, USA; 4 Alaska Colorectal Cancer Partnership, Anchorage, AK, USA

**Keywords:** storytelling, Alaska Native, telenovela, colorectal cancer screening, health communication, Community Health Workers

## Abstract

**Background:**

Alaska Native people have nearly twice the rate of colorectal cancer (CRC) incidence and mortality as the US White population.

**Objective:**

Building upon storytelling as a culturally respectful way to share information among Alaska Native people, a 25-minute telenovela-style movie, *What's the Big Deal?*, was developed to increase CRC screening awareness and knowledge, role-model CRC conversations, and support wellness choices.

**Design:**

Alaska Native cultural values of family, community, storytelling, and humor were woven into seven, 3–4 minute movie vignettes. Written post-movie viewing evaluations completed by 71.3% of viewers (305/428) were collected at several venues, including the premiere of the movie in the urban city of Anchorage at a local movie theater, seven rural Alaska community movie nights, and five cancer education trainings with Community Health Workers. Paper and pencil evaluations included check box and open-ended questions to learn participants' response to a telenovela-style movie.

**Results:**

On written-post movie viewing evaluations, viewers reported an increase in CRC knowledge and comfort with talking about recommended CRC screening exams. Notably, 81.6% of respondents (249/305) wrote positive intent to change behavior. Multiple responses included: 65% talking with family and friends about colon screening (162), 24% talking with their provider about colon screening (59), 31% having a colon screening (76), and 44% increasing physical activity (110).

**Conclusions:**

Written evaluations revealed the telenovela genre to be an innovative way to communicate colorectal cancer health messages with Alaska Native, American Indian, and Caucasian people both in an urban and rural setting to empower conversations and action related to colorectal cancer screening. Telenovela is a promising health communication tool to shift community norms by generating enthusiasm and conversations about the importance of having recommended colorectal cancer screening exams.

Prior to the mid-1900s, cancer was considered a rare disease among Alaska Native people, but since that time, cancer incidence and mortality have increased dramatically. For Alaska Native men and women combined, the average annual age-adjusted cancer incidence rate per 100,000 for the time period 1969–1973 was 370.7 and increased to 491.4 for the time period 2004–2008 (1). Colorectal cancer (CRC) is one of the most important contributors to the cancer burden among Alaska Native people. CRC is the second leading cause of cancer mortality among Alaska Native people, following lung cancer (1). Alaska Native people have nearly twice the rate of CRC incidence and mortality as the US White population. The age-adjusted CRC incidence rate (2004–2008) for Alaska Native men and women combined was 87.9 per 100,000 compared to the US White men and women combined rate of 45.1 per 100,000 (2004–2008) (1). The age-adjusted CRC mortality rate (2004–2008) for men and women combined was 30.2 per 100,000 for Alaska Native people and 16.6 per 100,000 for US Whites (2004–2008) (1). Completing recommended CRC screenings to find and remove precancerous polyps or to find and treat early cancers has the potential to contribute to the reduction of CRC incidence and mortality among Alaska Native people.

Culturally respectful ways of increasing CRC awareness and disseminating medically accurate screening information have the potential to empower Alaska Native people to make informed choices about their personal health behaviours. Alaska Native people have a rich heritage of using stories as a way to share knowledge and understanding. In the words of an Alaska Native elder,

Story is the way you talk, it is never direct on. Makes it easier to listen and hear. Makes it easier to talk about because you are not directly pointing the finger. In my tribe it is rude to point your finger; you did it with your chin or your eyebrow. So that's what I like about story. (Cueva, personal communication, 1 June, 2010)

Storytelling is a comfortable and natural way of giving and receiving information. Community Health Workers (CHWs) in Alaska have affirmed the ways storytelling supports their learning by facilitating knowledge and understanding and expanding perspectives (2).

Building upon storytelling as a culturally respectful way to share information among Alaska Native people, a 25-min CRC telenovela-style movie, *What's the Big Deal?* was developed in 2011 as an extension of the research-based Readers’ Theatre script with the same title (3). The original Readers’ Theatre script was developed with and for Alaska Native and American Indian CHWs and the people in their communities to provide CRC screening information, increase comfort with talking about CRC screening, and to encourage people to have recommended CRC screenings. Readers’ Theatre is the coming together of a group of people to read aloud a written conversation.

Telenovelas, which use serial dramatization to share socio-cultural messages, have been utilized as an effective health messaging strategy among Hispanic populations. Researchers found that a breast cancer storyline embedded within entertainment programming increased cancer knowledge and behavioural intent among Hispanic audiences (4). Similarly, a stroke and heart disease telenovela that incorporated cultural norms was found to increase knowledge between food and health (5). This project marks the first time a telenovela-style movie was developed, implemented and evaluated with and for Alaska Native people.

## Methods

The dynamic process of developing, implementing and evaluating this novel telenovela approach was informed by a socio-cultural approach to share health messages. Within this approach, cultural values, beliefs and behaviours are affirmed as a place of wisdom and resilience, and built upon to provide context and meaning for health messaging (6).

### Telenovela movie script

Woven throughout the telenovela script were Alaska Native people's cultural values and traditions reflecting the interconnected relationships of family, community, and the land (7). These interrelationships represent the core concept of the ecological model of public health, which acknowledges the interrelatedness of social, and community influences on health, as well as effects of genetics and personal behaviours (8, 9). Each serial storyline shares medically accurate information that serves to increase CRC screening awareness, support everyday conversations about colorectal screening, and encourage wellness choices that include colonoscopy screening. Key health messages identified by CHWs, cancer survivors, their families and caregivers and medical providers for the development of the evidenced-based Readers’ Theatre script (3) were included within the telenovela movie vignettes.

The movie includes seven, 3–4 min vignettes showing family and friends discussing screening while engaged in familiar activities such as eating together, playing cards, playing basketball and celebrating a 50th birthday, which is the recommended age for beginning CRC screenings. The storyline follows Rita, a young nursing student, as she educates and encourages her parents to have colorectal screening to support their health. At the end of each movie section, an engaging lead-in question, such as “Will Isaac call to make an appointment … Stay tuned” captures viewers’ curiosity about what happens next in the storyline.

By showing CRC screening conversations in a variety of social settings, this project sought to increase viewers’ comfort with talking about recommended CRC screening examinations. Character dialogue acknowledges the challenges of talking about CRC. For example, as Rita and her father, Isaac, eat breakfast, she shows him a colon health booklet to begin a challenging conversation about screening, and encourages him to read it. Isaac's response to his daughter's insistence is stated with humour: “This doesn't sound like something I want to talk about at the breakfast table.” As the conversation continues, Rita acknowledges colon health communication challenges, “There never seems to be a good time to talk about our colons.” and emphasizes the importance of family, “I love you and your health is important to me.”

### Anchorage movie premiere

The *What's the Big Deal?* telenovela was premiered at an Anchorage movie theatre on 15 August 2011. Invitations to the premiere were posted on the theatre website and posters were displayed in popular Anchorage locations, such as coffee shops and bingo halls.

### Rural Alaska community movie nights

The telenovela movie was shown in 7 different rural Alaska communities. Advertising included broadcasting announcements on the local radio as well as displaying posters in high-traffic areas, such as the Post Office, Laundromat, and health clinic.

### CHW movie showings

Five showings were conducted for CHWs as part of cancer education trainings. Telenovela showings provided an opportunity to increase CRC screening awareness and provide a health messaging tool for CHWs to share with their communities. Three CHW showings were held in Anchorage, Alaska and 2 in Tulsa, Oklahoma for American Indian Community Health Representatives (CHRs) as part of their national basic and refresher training.

### Written post-movie viewing evaluation tool design

Grounded within Freire's (10) adult education learning theory of empowerment, post-movie evaluation shifted from a pre-test/post-test approach to an integrated evaluation process to learn if and in what ways movie viewers may have internalized key health messages shared within the telenovela. By inviting viewers critical evaluation of the movie, we hoped to gain a deeper understanding of how and in what ways the serial telenovela movie may have influenced CRC knowledge and awareness, attitudes and perceptions about CRC screening, and ways cancer communication may have been enhanced.

The post-viewing written evaluation contained open-ended questions and yes/no check-box response questions. The evaluation card for the telenovela premiere had 4 questions to understand movie likeability and how the movie affected viewers’ CRC screening knowledge, attitudes towards CRC screening, and ways the movie may have influenced viewers’ intent to change health behaviours, particularly having colorectal screening. After the premiere, the evaluation form was expanded to a two-page written evaluation tool for both the community movie nights and CHW movie showings. The revised tool included additional questions that asked viewers about comfort talking about CRC screening, if they knew anyone diagnosed with CRC, if they had received a recommended CRC screening examination, as well as what factors influenced their decision to have screening. Information was collected on gender, age and ethnicity. Evaluation tools were collaboratively developed by the project team (article co-authors) and reviewed by interested health educators and CHWs for content readability as well as specific wording to elicit what we hoped to understand.

### Data analysis

All written evaluations from the telenovela premiere and the 12 telenovela showings were entered into an Excel database without personal identifiers. Qualitative data analysis of open-ended written evaluation responses was coded and analyzed by the project coordinator and reviewed by the project team for thematic consensus. Quantitative responses were analyzed using SPSS software. Descriptive statistics were calculated by demographic characteristics and responses to the evaluation questions.

## Results

The telenovela was shown and evaluated in 13 different venues in which 71.3% of viewers (305/428) completed a written post-movie viewing evaluation. From the 200 people present for the movie premiere, 103 (51.5%) written evaluations were collected. For this showing, viewer ethnicity was predominantly Caucasian (76.4%) with 12.3% of viewers being Alaska Native/American Indian. This was reflective of the 2011 United States Census Bureau demographics for Anchorage, 62% Caucasian and 8% Alaska Native/American Indian.

Between January and July of 2012, *What's the Big Deal?* was shown and evaluated during 7 community movie nights and 5 cancer education training sessions with CHWs in which 88.5% (202/228) of viewers completed a written evaluation. Community and CHW viewers were predominantly Alaska Native/American Indian (91.6%, 164/179) ( Table I).

**Table I:** Demographic characteristics of participants

	Anchorage premiere (1)	Community showings (7)	Community Health Worker showings (5)	Total 13 showings

	N (%) 103 (100%)	N (%) 114 (100%)	N (%) 88 (100%)	N (%) 305 (100%)
Gender				
Male	38 (40.0)	37 (37.4)	9 (11.3)	84 (31.0)
Female	57 (60.0)	62 (62.6)	71 (88.8)	190 (70.0)
Age				
49 and younger	59 (60.9)	41 (39.5)	57 (68.7)	157 (55.3)
50 and older	38 (39.2)	63 (60.5)	26 (31.3)	127 (44.8)
Ethnicity				
Alaska Native	9 (10.1)	90 (91.8)	26 (32.1)	125 (74.4)
American Indian	2 (2.2)	–	48 (59.3)	50 (18.7)
Caucasian	68 (76.4)	4 (4.1)	4 (4.9)	76 (28.4)
Others	10 (11.2)	4 (4.1)	3 (3.7)	17 (6.4)

### Anchorage movie premiere evaluation responses

In addition to the yes/no check-box responses, movie viewers wrote detailed comments about their movie experience (Table II).

**Table II:** Anchorage movie premiere question responses

Question	Yes	%
Is this movie an engaging/entertaining way to learn about colorectal screening?	101	98.1
After watching this movie, do you feel differently about colorectal screening?	64	62.1
Did this movie help you to learn more about colorectal screening?	78	75.7
After watching this movie, will you do anything differently in the ways you take care of your health?	73	70.9
• Talk with family and friends about colon screening	46	44.7
• Talk with my provider about colon screening	11	10.7
• Have a colon screening examination	18	17.5
• Increase my physical activity	30	29.1

N=103 total observations.

All respondents documented that the telenovela was an engaging and entertaining way to learn about colorectal screening. Viewers wrote that the telenovela relayed important health information in a “realistic and not too preachy” way. The storyline was described by viewers as being “engaging because it has a plotline with twists and a good ending” as well as being a “Great relatable story-real to Alaskans.”

Although the majority of movie premiere viewers in Anchorage were non-Native, they, as well as Alaska Native viewers, reported being able to relate with the characters in the movie at an emotional level: fear of screening, embarrassment to undergo the procedure and stubbornly resistant to screening, which drew them into the storyline and the lives of the characters. For example, several respondents specifically expressed concern for the character Beverly and wanted to learn the results of her colon polyp biopsy. In their evaluations, viewers highlighted the movie's effective use of humour, reporting that humour made the movie enjoyable to watch: “Great comic relief in an informative film.” Others commented that humour made it easier for viewers to hear the health messages without becoming defensive: “Emphasizes the importance [of CRC screening] in a fun entertaining way.”

Two-thirds of movie premiere respondents (64/97) described the ways they felt differently about colorectal screening after watching the telenovela. Viewer comments echoed prioritized key message objectives embedded within the telenovela script, such as normalizing recommended CRC screening: “It's no big deal [having CRC screening] - I like that message-it's a more normal human thing to do.” Additionally, viewers reported feeling more confident with their CRC knowledge, and motivated and empowered to talk about the importance of having recommended CRC screening examinations: “I feel like I can talk to my parents about it more confidently.” “We really need to talk about it [CRC screening] more. It's important.”

As a result of watching the telenovela, 79.5% (78/98) of the movie premiere respondents wrote information describing what they had learned including accurate colon health information which was shared throughout the storyline and ways to talk about CRC screening: “I learned how to talk to family about screening.”

Additionally, viewers repeatedly emphasized their hope that the telenovela would be shared with more people as emphasized in the following quotes: “Please make it available to as many clients as possible.” “Show it over and over; it was great!” “When is it going to air on TV?”

### Community and CHW evaluation responses

Of the 195 respondents who checked “yes, they would recommend this movie,” 112 (57.4%) wrote in-depth comments about why they would recommend this telenovela to others: “It might get someone to recognize how important it is to get screened. It should be watched by everyone so it [colorectal cancer] can be prevented or detected early” (Table III).

**Table III:** Community viewer and Community Health Worker (CHW) question responses

	Community viewers (N=114)	Community Health Worker viewers (N=88)	Total (N=202)

	Yes N (%)	Yes N (%)	Yes N (%)
Will you recommend this movie to others?	109 (95.6)	86 (97.7)	195 (96.5)
Did you like this movie?	110(96.5)	87 (98.9)	197 (97.5)
Is this movie a good way to learn about colorectal screening?	112 (98.2)	87 (98.9)	199 (98.5)
After watching this movie, do you feel differently about colorectal screening?	83 (72.8)	66 (75.0)	149 (73.8)
After watching this movie, do you feel more comfortable talking about colorectal cancer screening?	91 (79.8)	72 (81.8)	163 (80.7)
After watching this movie, will you do anything differently in the ways you take care of your health?	103 (90.4)	73 (83.0)	176 (87.1)
• Talk with family and friends about colon screening	62 (54.4)	54 (61.4)	116 (57.4)
• Talk with my provider about colon screening	28 (24.6)	20 (22.7)	48 (23.8)
• Have a colon screening examination	36 (31.6)	22 (25.0)	58 (28.7)
• Increase my physical activity	33 (28.9)	47 (53.4)	80 (39.6)
Do you know someone who has been diagnosed with colorectal cancer?	45 (39.5)	38 (43.2)	83 (41.1)
Have you had a colorectal screening examination?	38 (33.3)	26 (29.5)	64 (31.7)

N=total observations.

Of the 197 respondents who reported liking the movie, 122 (61.9%) wrote detailed descriptions of what they liked about the telenovela. Qualitative data analysis revealed several emergent themes, including the following topics. Viewers reported increased knowledge about CRC as a result of the movie: “I know more about colon cancer and I'm ready and going to get checked.” Additionally, they found the CRC messages to be simple and clear. “It was very educational and it wasn't full of medical terms, easy to follow. Simple to understand but shares the importance of CRC screening.” Another theme articulated how the movie engaged them in the storyline in an entertaining and enjoyable way: “Kept us involved-a fun way to learn.” Alaska Native Cultural values of humor, family, and storytelling were appreciated: “Humor lightened the subject.” “Very family-based movie, a lot of people can relate considering their own family.” “It used storytelling which helps you to hear the message.” Viewers felt hopeful as a result of the storyline: “Encourages others to get screened.”

Qualitative analysis of respondent written comments from both the movie premiere with a predominantly Caucasian audience and throughout Alaska with predominantly Alaska Native viewers revealed that movie viewers, regardless of ethnicity, described ways they related with the characters at an emotional level. “Able to relate to the stubbornness in the family. I would be like the Dad and want a support system to deal with the fear of testing. I'm always afraid to hear the truth, always in denial. There is nothing wrong with me, no reason why I should do the testing.” Consequently, an additional question was added to the evaluation tool inquiring if the viewers could relate to the characters in the movie. This question was included in the 2 movie showings with CHRs. These showings took place outside Alaska and with a predominantly American Indian audience of CHRs. Most (43, 78.2%) respondents reported that they were able to relate to the characters in this movie, 10 (18.2%) did not and 2 (3.6%) left the question blank.

Notably, 98.5% (199) of respondents noted that the telenovela-style movie was a good way to learn about colorectal screening. “It [telenovela-style] catches your attention more, keeps me more alert and awake.” “The humor aspects help with persuasion.” One hundred and three (51.7%) respondents described detailed information of how the movie supported their learning. Emergent themes included how the movie presented CRC facts and new information in engaging and humourous ways, respectfully honoured Alaska Native cultural values of family, community and humour as well as storytelling traditions, served as an ice-breaker to talk about the challenging topics of cancer, screening and colons, and role modeled colorectal-screening conversations among men and women of varying ages and with family, friends and health care providers. CRC information that viewers wrote, to describe what they learned, mirrored the key health messages that were prioritized to be included within the movie.

Most respondents (149/202, 73.7%) stated that they felt differently about colorectal screening after viewing the movie. Of the 48 viewers who reported not feeling differently, 34 wrote that they were already comfortable with CRC screening. Only 2 wrote ways they were still uncomfortable, “still is weird” and “not sure yet.” Additionally, 108 of the 149 respondents described how they felt differently, which included: emotional shifts categorized as less scary, less fearful, and more motivated, empowered and confident to have recommended CRC screening examinations or talk with others about having CRC screening examinations, as well as realizing how important having recommended CRC screening examinations are for personal, family and community health. “I feel more confident in taking the exam.” “I feel more encouraged to educate and to tell others about colorectal cancer.”

Of the 163 respondents who checked “yes that they felt more comfortable talking about CRC screening after watching the movie,” 102 (62.6%) wrote in-depth comments. Viewer comments illustrated how the movie supported their comfort with talking about CRC screening which they attributed to learning medically accurate CRC knowledge, feeling more comfortable with CRC screening procedures, and experiencing ways that people successfully talked about CRC screening in the movie, hearing the words they used. In the words of respondents, “After watching each chapter of the movie, I feel more prepared with information I might need to answer questions and feel more comfortable approaching family members and friends. If someone asks me, ‘What's your colon?’ I now know.” “I feel I learned a lot of information and I will be able to explain it better.” “It's normalized. I feel comfortable enough to make an appointment for myself.” “It's a normal thing, everyone should be getting it done. The information in the movie helps with overcoming being anxious. No big deal!”

Of the 202 study participants, 64 (31.7%) reported having had a colorectal screening. Of the 64, 50 (78%) were aged 50 and older. Of the 39 respondents, aged 50 and older, who had not had a recommended CRC screening examination, 36 wrote of intentions to change behaviour. Of the 36, 21 (58%) planned to have a CRC screening examination, 13 (36%) planned to talk with their health care provider about colon screening, 21 (58%) planned to talk with family and friends about colon screening, and 15 (42%) wrote about their intent to increase physical activity.

## Discussion

The reactions by viewers of the telenovela-style movie *What's the Big Deal?* strongly suggest that it was considered to be an engaging way to share medically accurate CRC information to increase viewers’ knowledge and role model ways to talk about CRC screening. Similarly, telenovelas were found to appeal to multi-generations within the Hispanic population and were often viewed together to maintain family bonds and provide inspiration and self-affirmation as the movie characters surmounted situations and overcame their fears (11).

Movie evaluations indicated that the telenovela storyline and characters connected with people across cultures, as it spoke to people at an affective place of knowing. Through the narrative nature of the movie, viewers described being emotionally engaged with the characters and the storyline. Data analysis revealed that both Native and non-Native viewers responded positively to the movie. Among both groups, over two-thirds reported an intention to improve their health. As a result of movie viewing, people described feeling differently about their health choices including written plans to have recommended CRC screening examinations, talking with their health care provider about CRC screenings, recommending and encouraging family and friends to have CRC screenings, and increasing their physical activity. Watching the movie provided the motivation and language to empower viewers who were either too young to have recommended CRC screenings or who already had CRC screenings to pass the message forward to family and friends. In the representative words of a movie viewer, “It is so hard to talk about cancer. This movie helps us to open up and start conversations about colorectal cancer and getting screened in an entertaining, easy to watch way. Drives the message home with humor.”

## Conclusion

Through this project, the telenovela movie genre was introduced to Alaska Native people and was found to be an engaging CRC health messaging tool. Viewers reported an increase in their CRC knowledge and comfort with talking about CRC screenings. Of note, 81.6% of respondents (249/305) wrote about their positive intent to change behaviour. Written evaluations revealed the telenovela genre to be an innovative way to communicate CRC health messages with Alaska Native, American Indian and Caucasian people both in an urban and rural setting to empower conversations and action related to CRC screening. Telenovela is a promising health communication tool to shift community norms by generating enthusiasm and conversations about the importance of having recommended CRC screening examinations.

Due to movie likeability, the telenovela has been widely distributed in Alaska and beyond. The movie is shown in the waiting areas of the Alaska Native Primary Care Center in Anchorage and Alaska's rural village clinics. Additionally, this telenovela can be viewed on YouTube. As of June 2013, the YouTube website http://www.youtube.com/watch?v=2DPgnlirW5M had been accessed 675 times by people from 25 different countries. The telenovela is also posted on the Community Health Aide Program cancer education web page at akchap.org. During 2012 over 500 copies of the telenovela DVD were distributed upon request to Alaska's CHWs and health educators. Additionally, Native CIRCLE, a national clearinghouse for resources for Indigenous people, distributed over 400 DVDs in response to online and telephone requests and conference displays.
